# Added value of dynamic contrast-enhanced MR imaging in deep learning-based prediction of local recurrence in grade 4 adult-type diffuse gliomas patients

**DOI:** 10.1038/s41598-024-52841-7

**Published:** 2024-01-25

**Authors:** Jungbin Yoon, Nayeon Baek, Roh-Eul Yoo, Seung Hong Choi, Tae Min Kim, Chul-Kee Park, Sung-Hye Park, Jae-Kyung Won, Joo Ho Lee, Soon Tae Lee, Kyu Sung Choi, Ji Ye Lee, Inpyeong Hwang, Koung Mi Kang, Tae Jin Yun

**Affiliations:** 1 Department of Radiology, Seoul National University College of Medicine, 101, Daehangno, Jongno-gu, Seoul, 03080 Republic of Korea; 2 Department of Radiology, Seoul National University Hospital, Seoul, Republic of Korea; 3 Center for Nanoparticle Research, Institute for Basic Science (IBS), Seoul, Republic of Korea; 4 School of Chemical and Biological Engineering, Seoul National University, 1, Gwanak-ro, Gwanak-gu, Seoul, 302-909 Republic of Korea; 5 Department of Internal Medicine, Cancer Research Institute, Seoul National University College of Medicine, Seoul, Republic of Korea; 6 Department of Neurosurgery, Biomedical Research Institute, Seoul National University College of Medicine, Seoul, Republic of Korea; 7 Department of Pathology, Seoul National University College of Medicine, Seoul, Republic of Korea; 8 Department of Radiation Oncology, Cancer Research Institute, Seoul National University College of Medicine, Seoul, Republic of Korea; 9 Department of Neurology, Seoul National University College of Medicine, Seoul, Republic of Korea

**Keywords:** Oncology, CNS cancer

## Abstract

Local recurrences in patients with grade 4 adult-type diffuse gliomas mostly occur within residual non-enhancing T2 hyperintensity areas after surgical resection. Unfortunately, it is challenging to distinguish non-enhancing tumors from edema in the non-enhancing T2 hyperintensity areas using conventional MRI alone. Quantitative DCE MRI parameters such as K^trans^ and V_e_ convey permeability information of glioblastomas that cannot be provided by conventional MRI. We used the publicly available nnU-Net to train a deep learning model that incorporated both conventional and DCE MRI to detect the subtle difference in vessel leakiness due to neoangiogenesis between the non-recurrence area and the local recurrence area, which contains a higher proportion of high-grade glioma cells. We found that the addition of V_e_ doubled the sensitivity while nonsignificantly decreasing the specificity for prediction of local recurrence in glioblastomas, which implies that the combined model may result in fewer missed cases of local recurrence. The deep learning model predictive of local recurrence may enable risk-adapted radiotherapy planning in patients with grade 4 adult-type diffuse gliomas.

## Introduction

Grade 4 adult-type diffuse glioma is an aggressive malignant brain tumor that has a high recurrence rate, and the median survival remains at only 15 months^1,2^. The standard treatment for grade 4 adult-type diffuse glioma is a multimodality strategy including maximal surgical resection followed by radiation therapy with concurrent temozolomide (TMZ) and adjuvant TMZ^2^. Given that the goal of glioma surgery is to maximally remove the tumor while preserving the patient’s functional integrity, the main target for surgical resection is often limited to contrast-enhancing tumor components on contrast-enhanced T1-weighted images (CE T1WIs), and non-enhancing tumor components intermingled with peritumoral edema inevitably remain after surgical resection^1,3,4^. It has been recognized that local recurrence occurs at residual non-enhancing lesions and affects the prognosis of grade 4 adult-type diffuse glioma patients^5^. Unfortunately, it is challenging to distinguish the non-enhancing tumor infiltration from edema because they are similarly presented as lesions with high signal intensity on T2-weighted or T2 fluid-attenuated inversion recovery (FLAIR) images on conventional MR imaging^6,7^.

Therefore, much effort has been focused on the use of advanced MR imaging for identification of non-enhancing tumor cells that are not distinguishable on conventional MR imaging. Dynamic contrast-enhanced (DCE) MR imaging has emerged as a promising method for quantifying microvascular permeability^8–12^. The important pharmacokinetic parameters of DCE MR imaging are the volume transfer constant (K^trans^), extravascular extracellular space per unit volume of tissue map (V_e_), and blood plasma volume per unit volume of tissue map (V_p_)^8–12^. Prior studies have recognized that baseline and posttreatment DCE MR imaging parameters of non-enhancing T2 high signal intensity lesions have prognostic values and predict the recurrence in grade 4 adult-type diffuse glioma patients undergoing the standard treatment^7,13–15^.

Recently, several studies have built machine learning or deep learning based local recurrence and survival prediction models that use radiomic features of conventional and advanced MR imaging parameters such as apparent diffusion coefficient (ADC), diffusion tensor imaging, and cerebral blood volume (CBV)^16–22^. However, to our knowledge, there have been no reports on the use of deep learning of multiparametric MR imaging, including conventional and DCE MR imaging, for predicting local recurrence in grade 4 adult-type diffuse gliomas. Deep learning techniques, when used in conjunction with multiparametric MR imaging, may be helpful for predicting local recurrence in grade 4 adult-type diffuse glioma patients, given its clear advantage in processing complex imaging data^23^. Hence, the purpose of our study was to develop a multiparametric deep learning model based on DCE and conventional MR imaging for prediction of local recurrence in patients with grade 4 adult-type diffuse gliomas.

## Results

### Patient characteristics

The clinical characteristics of the recurrence and non-recurrence groups in the total study population are summarized in Supplementary Table 1. There were no differences between the two groups with regard to age and sex (*P* = 1.44 and *P* = 0.13, respectively). The incidence of IDH wildtype was significantly higher in the recurrence group (97% [75 of 77]) than in the non-recurrence group (89% [91 of 102]) (*P* = 0.04). The incidence of promoter methylation of MGMT was significantly higher in the non-recurrence group (71% [77 of 102]) than in the recurrence group (27% [21 of 77]) (*P* < 0.001). The clinical characteristics of the patients in the training and test sets are summarized in Table 1.

**Table 1:** Clinical characteristics of the training and test sets.

Characteristics	Total (n = 179)	Training set (n = 146)	Test set (n = 33)	*P* value
Mean age (years)*	57.7 ± 13.4	57.1 ± 13.7	60.5 ± 11.9	0.19^†^
Sex				1.00^‡^
Male	92 (51)	75 (51)	17 (52)	
Female	87 (49)	71 (49)	16 (48)	
Methylated MGMT promoter				0.85^‡^
Positive	98 (55)	79 (54)	19 (58)	
Negative	81 (45)	67 (46)	14 (42)	
IDH1/2 mutation				0.57^‡^
Positive	13 (7)	12 (8)	1 (3)	
Negative	165 (92)	133 (91)	32 (97)	
Not available	1 (1)	1 (1)	0 (0)	

Unless otherwise indicated, data represent the number of patients (percentages).

MGMT = O^6^-methylguanine-DNA methyltransferase, IDH = isocitrate dehydrogenase.

*Data are means ± SD.

^†^Calculated with the independent samples t-test.

^‡^Calculated with Fisher’s exact test.

### Diagnostic performance of deep learning models

**Figure 1:**
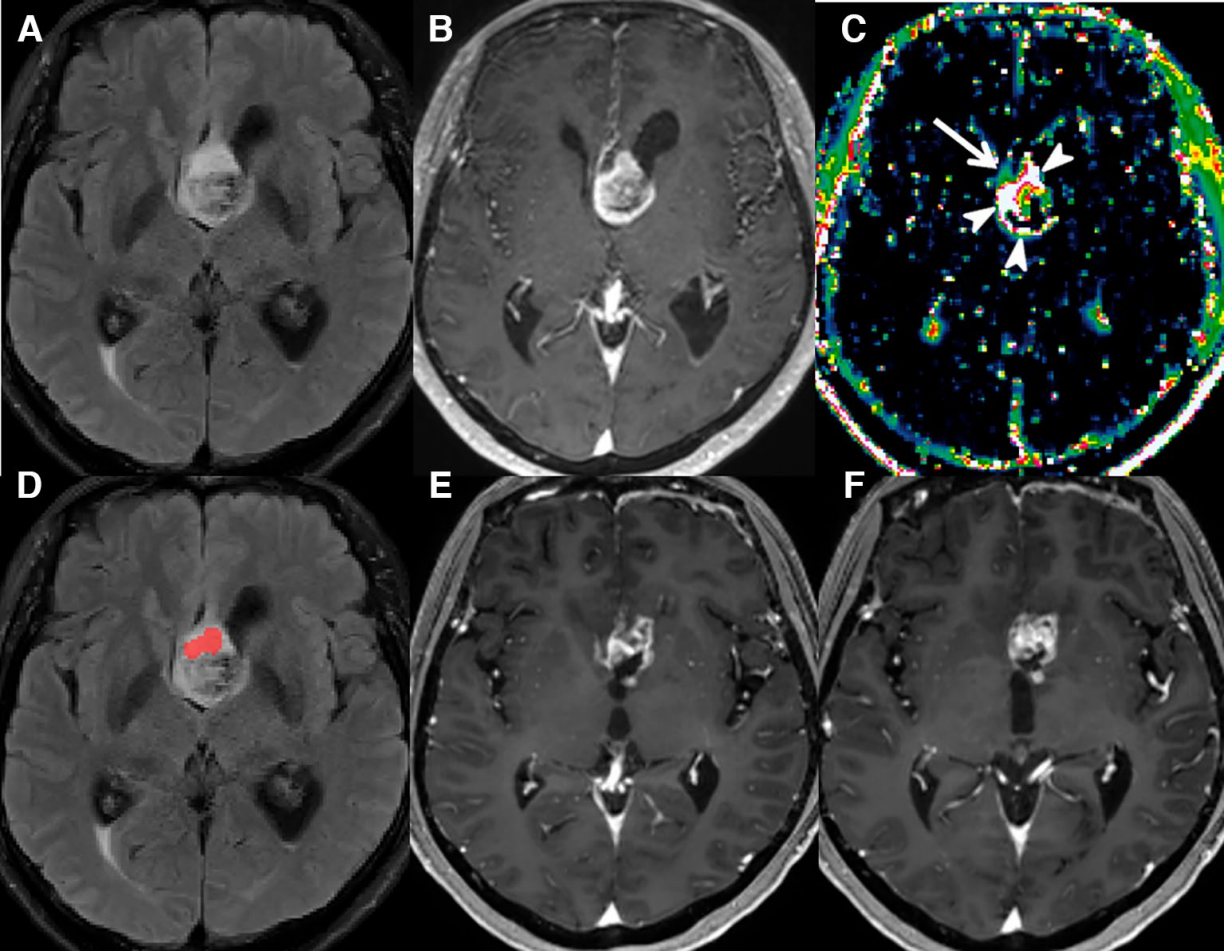
A 60-year-old woman with GBM in the training set. (**A**,**B**) Preoperative T2 FLAIR and CE T1W images show a heterogeneously enhancing mass at the septum pellucidum and corpus callosum with a small surrounding area of non-enhancing T2 hyperintensity. (**C**) The V_e_ map depicts a mild increase at the anterior aspect of the enhancing tumor (arrow) along with an overt increase at the enhancing portion of the tumor (arrowheads). (**D**) The combined MR model predicted local recurrence to occur at the non-enhancing T2 hyperintense lesion anterior to the enhancing tumor (red area). (**E**,**F**) At the 7-month follow-up, a measurable enhancing lesion appeared at the corpus callosum (genu). *GBM* glioblastoma, *FLAIR* fluid-attenuated inversion recovery, *CE T1W* contrast-enhanced T1-weighted.

**Figure 2:**
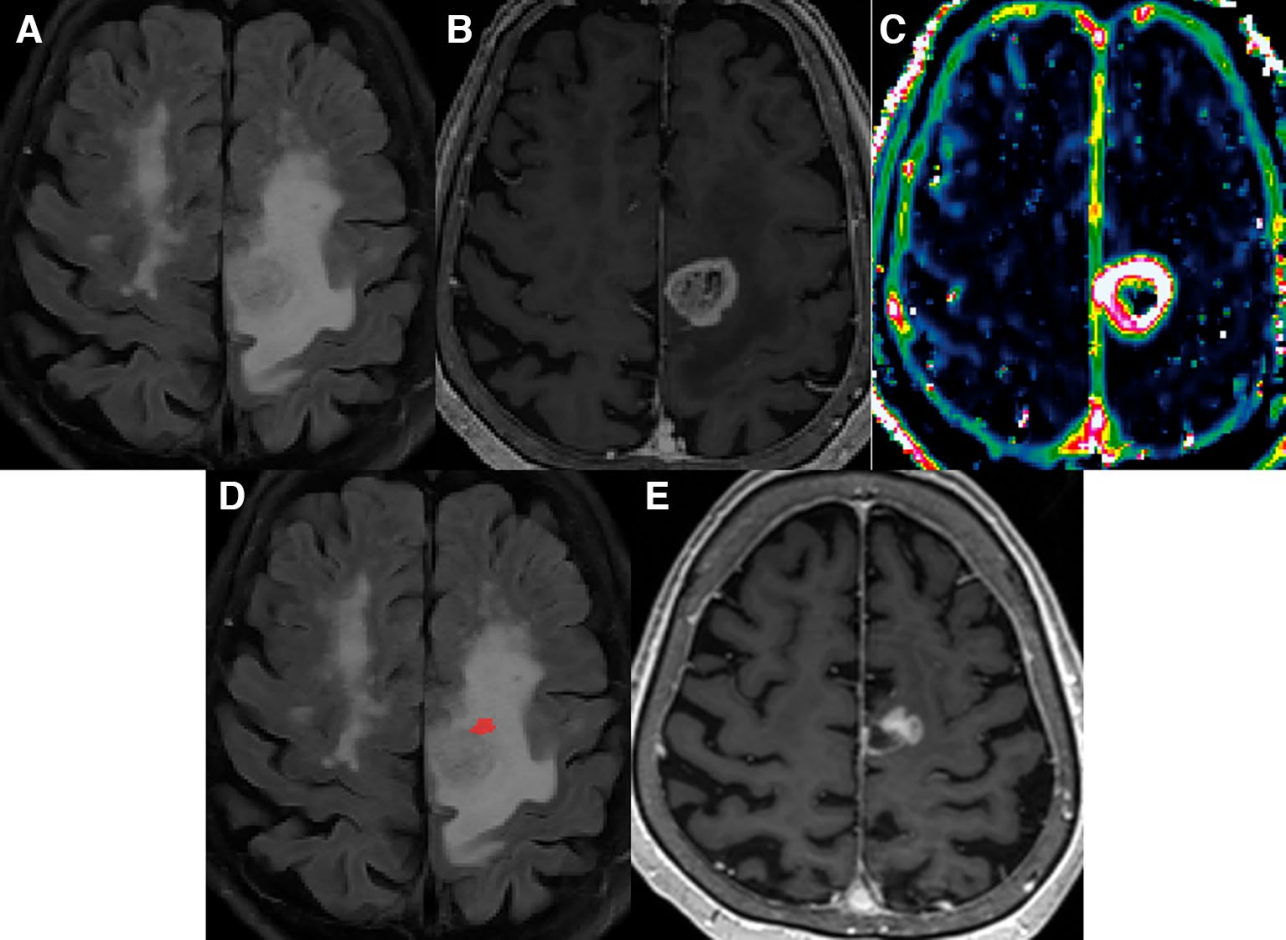
A 71-year-old woman with GBM in the test set. (**A**,**B**) Preoperative T2 FLAIR and CE T1W images demonstrate a heterogeneously enhancing mass at the left parietal lobe with extensive perilesional T2 hyperintensity. (**C**) The V_e_ map depicts an overt increase at the enhancing portion of the tumor with no discernible change at the non-enhancing T2 hyperintense portion. (**D**) The combined MR model predicted local recurrence to occur at the anterior aspect of the enhancing tumor (red area). On the other hand, recurring voxels were predicted to be ‘absent’ according to the conventional model. (**E**) At the 7-month follow-up, a measurable enhancing lesion appeared at the anterior aspect of the surgical cavity. *GBM* glioblastoma, *FLAIR* fluid-attenuated inversion recovery, *CE T1W* contrast-enhanced T1-weighted.

In the training set, the sensitivity for predicting local recurrence was higher in the combined MR model than in the conventional MR model (69% [42 of 61; 95% CI 56, 80] vs. 59% [36 of 61; 95% CI 46, 71]; *P* = 0.33), although statistical significance was not reached. There was no statistically significant difference in the specificity for predicting local recurrence between the combined MR model and conventional MR model (34% [29 of 85; 95% CI 24, 45] vs. 31% [26 of 85; 95% CI 21, 42], respectively; *P* = 0.69). In the test set, the combined MR model showed a significantly higher sensitivity (80% [12 of 15; 95% CI 52, 96]) than the model based on conventional MR imaging alone (40% [6 of 15; 95% CI 16, 68]) (*P* = 0.03) for prediction of local recurrence. The specificity was not significantly different between the combined MR model and the conventional MR model (44% [8 of 18; 95% CI 22, 69] vs. 50% [9 of 18; 95% CI 26, 74], respectively; *P* = 1.00). The representative cases of local recurrence prediction in the training and test sets are shown in Figs. 1, 2.

## Discussion

In this study, we explored the potential of two nnU-Net based deep learning models for prediction of local recurrence in patients with grade 4 adult-type diffuse gliomas. The model based on the combination of conventional MR imaging and V_e_ map from DCE MR imaging had a higher sensitivity than that based on conventional MR imaging alone at a similar specificity for prediction of local recurrence in grade 4 adult-type diffuse gliomas.

Non-enhancing T2 hyperintense lesions of grade 4 adult-type diffuse gliomas are a mixture of infiltrative tumor cells and peritumoral edema. The interface between contrast-enhancing tumors and non-enhancing tumors is of clinical significance considering that most of the local recurrence occurs at the resection margin. DCE MR imaging depicts the permeability characteristics of the tumor that cannot be provided by conventional MR imaging, with quantitative parameters such as K^trans^ and V_e_ reflecting the exchange between the vasculature in the tissue and interstitium and leakage space^15^. Active neoangiogenesis and vessel leakiness are the hallmarks of high-grade gliomas and K^trans^ and V_e_ values have been reported to be higher in high-grade gliomas than in low-grade gliomas^24^. Specifically, the structural basis of the unusual leakiness in vessels of high-grade tumors has been attributed to openings between defective endothelial cells characterized by disorganized, loosely connected, branched, overlapping or sprouting configurations^25^. We speculated that the local recurrence area would have a higher proportion of high-grade tumor component among the non-enhancing T2 hyperintense lesion, which could be reflected on V_e_ maps, but not on conventional MR images including T2 FLAIR and CE T1WI. Our results are in agreement with those of the previous study, in which V_e_ was increased in recurring voxels within the white matter and gray matter of the gross tumor volume delineated on RT-MRI, as compared with non-recurring voxels^26^. Pak et al. also reported that the radiomics risk score calculated from 16 features, including 9 features from the V_e_ map of the non-enhancing T2 high signal intensity region, was associated with progression-free survival independent of IDH mutation status^15^. Unlike the previous study, we focused on the small local recurrence area among the non-enhancing T2 hyperintense lesion. Since local recurrence first occurs in small clusters of high-grade glioma cells with most representative high-grade features, we speculated that using a small label confined to a small local recurrence area in the earliest possible time would have a higher analytic value than using a mask encompassing the entire non-enhancing T2 hyperintense lesion or a larger local recurrence area at the later time.

Enhancing tumor components of grade 4 adult-type diffuse gliomas with high cell densities are likely to cause overt changes in relative CBV and permeability^10,27^. In contrast, changes in the perfusion and permeability parameters attributable to infiltrative high-grade tumors hidden among non-enhancing T2 hyperintense lesions are relatively subtle such that it is often challenging to distinguish infiltrative high-grade tumors with mildly increased V_e_ from peritumoral edema with our human eyes. Machine or deep learning algorithms capable of processing hidden information have been suggested as powerful assistive tools for otherwise very challenging or impossible classification or prediction tasks to the unassisted eyes^28–31^. Based on radiomics features extracted from diffusion tensor imaging and relative CBV maps, Rathore et al. used a machine learning algorithm, a support vector machine classifier, to develop a model predictive of local recurrence, by identifying the relatively more-infiltrated regions with higher cellularity and perfusion among non-enhancing T2 high signal intensity regions^21^. In this study, we were able to build a prediction model based on the difference in permeability characteristics between the local recurrence and non-recurrence areas using the nnU-Net algorithm.

At present, clinical tumor volume in radiotherapy planning often encompasses the non-enhancing T2 hyperintense lesion, given that the non-enhancing T2 hyperintense lesion includes not only peritumoral edema but also non-enhancing tumor components due to the infiltrative nature of grade 4 adult-type diffuse gliomas. In terms of clinical implications, we speculate that the deep learning model may have a potential role in risk-adapted RT planning in the future, because a higher radiation dose may be delivered to a broader area in patients predicted to have local recurrence by the model to achieve better local control. Regarding the diagnostic performance of deep learning models, the addition of V_e_ doubled the sensitivity while decreasing the specificity nonsignificantly, which implies that the combined model would result in fewer cases of missed local recurrence and more cases of non-recurrence receiving unnecessarily high radiation dose. Although unnecessarily high radiation dose can lead to increased radiation-induced complications, we speculate that the clinical consequence of missing local recurrence in patients who could potentially have survival gain from higher radiation dose would be greater in the clinical setting. Furthermore, considering the high incidence of early local recurrence in adult-type diffuse glioma (grade 4), we anticipate that employing a deep learning algorithm characterized by increased sensitivity (albeit reduced specificity) would yield greater clinical advantages.

Our study has several limitations. First, this was a retrospective study based on a relatively small study population, and thus, the results could have been influenced by selection bias. Nonetheless, by using randomly generating patches with various data augmentation techniques such as cropping and rotation for learning, the model was trained to prepare for various inputs even with a small dataset. Second, the model was validated in patients sampled at a later time point to provide some information on the generalizability as well as the reproducibility of our prediction model without external validation. A future prospective multicenter study is warranted to provide stronger evidence for the generalizability of the model. Third, not all patients from the study period had V_e_ maps available for analysis due to incomplete datasets or suboptimal image quality of DCE MR imaging. Fourth, to simplify the deep learning prediction models as in a previous study^32^, local recurrence was analyzed as a binary measure (the presence of local recurrence at 1 year) without considering time information such as time to progression. Given the time-dependent nature of recurrence data, the deep learning prediction model may be refined by incorporating the time information in the future. Fifth, the exact localization of local recurrence areas on the preoperative FLAIR images was challenging in some cases due to anatomical distortion after surgery. We made efforts to minimize the mismatch between annotated labels on preoperative FLAIR images and true local recurrence areas on follow-up MR images by taking into account anatomical relationships with surrounding structures and by cross-checking the labels with expert radiologists with more than 12 years of experience. Sixth, although the prognosis varies among ‘grade 4 adult-type diffuse gliomas’ according to the IDH mutation status^33–38^, we grouped them together to develop the deep learning models in this study. Grade 4 adult-type diffuse gliomas are characterized by their infiltrative growth, and therefore, have infiltrative tumor cells within the non-enhancing T2 hyperintensity regardless of the IDH mutation status^39^. Moreover, the standard treatment strategies are also the same for both ‘glioblastoma, IDH-wildtype, grade 4’ and ‘astrocytoma, IDH-mutant, grade 4’ at present^40^. Seventh, the sensitivity of the combined model was higher in the test set, as compared with the training set. The inconsistent sensitivity results between the two datasets may be possibly attributed to several factors including the following: 1) The possibility exists that the test data happened to be drawn more favorably for the combined MR model by chance; 2) The test data consisted of more recent data with relatively higher spatial and temporal solutions for DCE MR imaging. A future prospective study based on a larger dataset is needed to validate the added value of V_e_ information for deep learning-based prediction of local recurrence in grade 4 adult-type diffuse gliomas.

In conclusion, a multiparametric nnU-Net deep learning model based on the combination of DCE and conventional MR imaging outperformed the nnU-Net model based on conventional imaging alone in terms of sensitivity for prediction of local recurrence in patients with grade 4 adult-type diffuse gliomas. The model output may be used to modulate the radiation dose to achieve better local control and ultimately improve the median survival in patients with grade 4 adult-type diffuse gliomas.

## Methods

The institutional review board of Seoul National University Hospital approved this retrospective study and waived the requirement for informed consent (IRB No. 2111-191-1277). The study protocol is performed in accordance with with the Declaration of Helsinki.

### Patient selection

**Figure 3:**
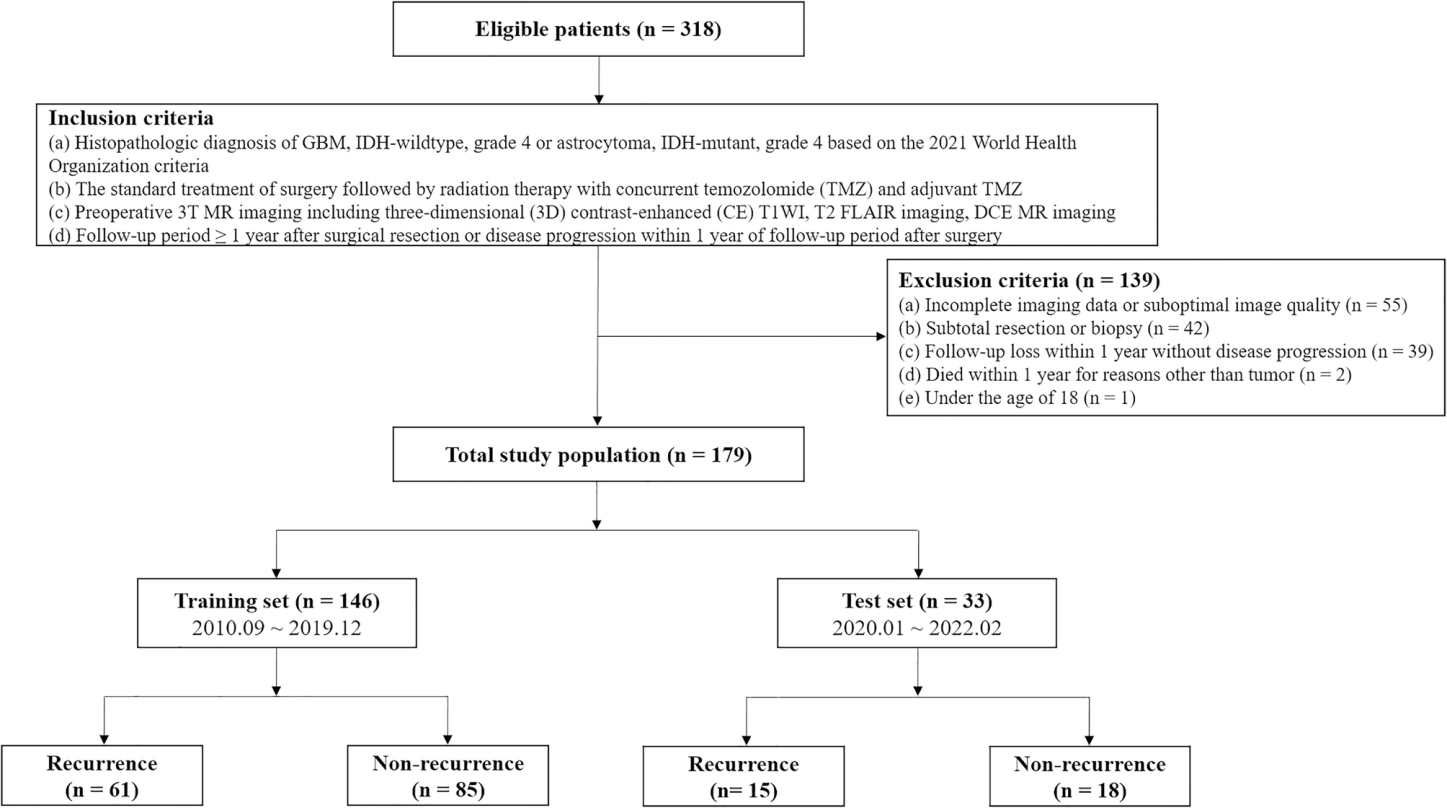
Flow diagram of patient selection and classification. The training set consisted of 62 recurrence and 84 non-recurrence cases, and the test set consisted of 15 recurrence and 18 non-recurrence cases.

From September 2010 to February 2022, 318 patients who were initially diagnosed with GBM, isocitrate dehydrogenase (IDH)-wildtype, grade 4 or astrocytoma, IDH-mutant, grade 4 at Seoul National University Hospital were consecutively enrolled (Fig. 3).

The inclusion criteria were as follows: patients (a) who had a histopathologic diagnosis of GBM, IDH-wildtype, grade 4 or astrocytoma, IDH-mutant, grade 4 based on the 2021 World Health Organization (WHO) criteria^41^; (b) who underwent the standard treatment of surgery followed by radiation therapy with concurrent TMZ and adjuvant TMZ medication; (c) who underwent preoperative 3 T MR imaging including three-dimensional (3D) CE T1WI, T2 FLAIR, and DCE MR imaging; and (d) who had a follow-up period ≥ 1 year after surgery or who were considered to have disease progression within 1 year of the postoperative follow-up period.

The exclusion criteria were as follows: patients (a) who had incomplete imaging data or suboptimal image quality (n = 55); (b) who underwent subtotal resection or biopsy (n = 42); (c) who were lost to follow-up within 1 year without diagnosis of disease progression (n = 39); (d) who died within 1 year for reasons other than tumor (n = 2); and (e) who were under the age of 18 (n = 1).

As a result, a total of 179 patients who received gross total resection of contrast-enhancing lesions (i.e., no residual contrast-enhancing lesions other than postoperative change on immediate postoperative MR imaging) were finally included in our study. Of the 179 total study participants, 146 patients who were diagnosed between 2010 and 2019 were allocated to a training set to develop a model. This model was further evaluated on a test set consisting of 33 patients who were diagnosed between 2020 and 2022.

Furthermore, our dataset was divided into a recurrence group (n = 77) and a non-recurrence group (n = 102). The training set consisted of 62 recurrence and 84 non-recurrence cases, and the test set consisted of 15 recurrence and 18 non-recurrence cases. In this study, we defined the ‘local recurrence’ within 1 year after surgery as the appearance of a measurable enhancing lesion located within non-enhancing T2 hyperintense area around the surgical cavity^42^. The flow diagram of patient selection and classification is shown in Fig. 3. For all patients, clinical characteristics were recorded, including age, sex, methylation status of O6-methylguanine-DNA methyltransferase (MGMT) promoter, and IDH mutation status.

### MR image acquisition

All preoperative MR images were obtained using a 3 T imaging unit with a 32-channel head coil (Magnetom Verio [n = 100], Siemens Healthineers; Magnetom Skyra [n = 63], Siemens Healthineers; Ingenia CX 3.0 T [n = 15], Philips Healthcare; Discovery MR 750w [n = 1], GE Healthcare). The MR imaging protocol for the tumor evaluation included the following sequences: pre- and postcontrast T1WI (T1-weighted 3D magnetization-prepared rapid acquisition gradient-echo [MPRAGE] sequence), axial T2 FLAIR imaging, and DCE MR imaging.

For DCE MR imaging, 3D T1-weighted spoiled gradient-echo imaging was performed. Gadobutrol (Gadovist; Bayer Schering Pharma, Berlin, Germany) (0.1 mmoL/kg of body weight) and a 30 mL saline bolus were injected at a rate of 4 mL/s, using a power injector (Spectris; MedRad, Indianola, Pennsylvania). For each phase, 40 images were acquired at intervals of repetition time (TR) with a temporal resolution of 4.8 to 6 s. The specific parameters were as follows: TR = 2.8–4.2 ms; echo time (TE) = 1.0–2.1 ms; flip angle = 10°; matrix = 192 × 192 or 128 × 128; field of view (FOV) = 240 × 240 mm^2^; section thickness = 3.0 mm; voxel size = 1.25 × 1.25 × 3 mm^3^ or 1.87 × 1.87 × 3 mm^3^; pixel bandwidth = 543–790 Hz; phase = 60; and total acquisition time = 4 min 58 s or 5 min 5 s. The MR scan parameters of all sequences are summarized in Supplementary Table 2.

### Image analysis of DCE MR imaging

Postprocessing of DCE MR imaging was performed with commercial software (Nordic ICE, v4.1.2; NordicNeuroLab, Bergen, Norway). The two-compartment pharmacokinetic model proposed by Tofts and Kermode was used to calculate the extravascular extracellular space volume per unit volume of tissue (V_e_) from DCE MR imaging^43^. To generate DCE parameter maps, vascular deconvolution with the arterial input function (AIF) was carried out. For each tumor, one experienced neuroradiologist (R.E.Y. with 12 years of neuro-oncology imaging experience), who was blinded to the prognosis information, determined the AIF at the M1 segments of the middle cerebral arteries (at the level of the Circle of Willis). The final AIF curve was made using the cluster analysis technique. The baseline T1 was fixed at 1000 ms in this study^44^.

### Development of the deep learning models

#### Annotation of local recurrence on preoperative MR imaging

All preoperative MR images in the training and test data were manually labelled by investigators supervised by two expert radiologists (R.E.Y. and S.H.C. with 12 and 19 years of neuro-oncology imaging experience) using the ITK-SNAP software tool (v3.8.0; http://www.itksnap.org)^45^. Using the follow-up CE T1WIs at the time of recurrence as the reference standard, two investigators, by consensus, carefully defined the regions of interest (ROIs), the local recurrence areas, within every section of a non-enhancing T2 hyperintense lesion on the preoperative FLAIR images (Supplementary Fig. 1A). The investigators were blinded to DCE MR imaging when they performed manual labeling of local recurrence on preoperative FLAIR images.

#### Image processing

3D CE T1WIs were resampled to a 1 mm isovoxel with linear interpolation. Subsequently, the following images were coregistered and resampled to the corresponding 3D 1 mm isovoxel CE T1WIs using BRAINSFit of 3D Slicer: preoperative 2D FLAIR images, manual annotation labels on the FLAIR images, and 2D DCE parameter (V_e_) maps (Supplementary Fig. 1A). Among various DCE parameters, we chose to focus on V_e_ map for our deep learning model development based on a previous study^15^, which reported that V_e_ related radiomics features from non-enhancing T2 high SI region contributed the most to the radiomics risk score for predicting the progression-free survival in GBM patients. All coregistration results were manually checked and confirmed by two expert radiologists (R.E.Y. and S.H.C. with 12 and 19 years of neuro-oncology imaging experience).

#### Deep learning model development

The local recurrence prediction models were developed using nnU-Net (Supplementary Fig. 1B)^46^. As the name implies, nnU-Net uses the same neural network design as U-Net, but nnU-Net focuses on pre/post data processing and hyperparameter setting for higher performance and practicability. A recent investigation has shown that nnU-Net exhibited superior performance, as compared with the majority of existing methods, across various tasks on 23 publicly available datasets in international biomedical segmentation competitions^46^. Notably, nnU-Net has several advantages: (a) it autonomously adapts to new tasks, encompassing preprocessing, network architecture, training, and post-processing; (b) it accommodates a diverse range of biomedical imaging datasets; (c) it operates without the need for user intervention; and (d) it is computationally viable.

In the model training, 3D CE T1WI and FLAIR pairs were fed as input to build the conventional MR model while 3D CE T1WI, FLAIR, and V_e_ map datasets were fed as input to build the combined MR model. Subsequently, nnU-Net automatically determined the hyperparameters related to the model training in consideration of the core characteristics of the dataset (dataset fingerprint), including the class ratio image size and the voxel spacing information (details are provided in ‘Supplementary Materials’, Supplementary Table 3, and Supplementary Fig. 2).

We trained full resolution 3D models rather than using 2D models or cascade approaches because 3D models are expected to perform better than 2D models, which have been shown to predict outcomes based on limited information as compared with 3D models. It was easier to obtain working deep learning models since nnU-Net covered data augmentation, patch generation, and patch stitching to yield final prediction results.

### Statistical analysis

All statistical analyses were performed using statistical software (MedCalc, version 11.1.1.0, Mariakerke, Belgium). The data for each parameter were assessed for normality with the Kolmogorov–Smirnov test. The clinical characteristics of the recurrence and non-recurrence groups were compared using the independent samples t-test for non-categorical variables and the Fisher’s exact test for categorical variables. The McNemar test was used to compare the sensitivity and specificity between the conventional MR model and the combined MR model based on both conventional and DCE MR imaging (V_e_ map). The model output included voxels with the probability of ‘0.5’. For calculation of the sensitivity and specificity at the patient level, cases with at least one voxel in the predicted results were categorized as ‘local recurrence’. In all tests, P values less than 0.05 were considered statistically significant.

### Supplementary Information

Supplementary Information.

## Data Availability

The datasets generated during and/or analysed during the current study are available from the corresponding author on reasonable request.

## Code availability

To train the deep learning model in this study, we used the publicly available nnU-Net architecture available at https://github.com/MIC-DKFZ/nnUNet.
