# Supplementary material for: Added value of dynamic contrast-enhanced MR imaging in deep learning-based prediction of local recurrence in grade 4 adult-type diffuse gliomas patients
Source: Sci Rep. 2024 Jan 25;14:2171. doi: 10.1038/s41598-024-52841-7 (PMC10810891; doi:10.1038/s41598-024-52841-7)
Supplement: Supplementary file 1 — Supplementary Information. [file 41598_2024_52841_MOESM1_ESM.docx]

**Supplementary Materials**

*Deep Learning Model Development*

The architecture of nnU-Net is based on the U-Net architecture, which includes an encoder and a decoder connected by a series of skip connections. The encoder comprises convolutional and pooling layers that progressively downsample the input image, while the decoder pathway uses upconvolutional and concatenation operations to gradually upsample the feature maps and recover the original image resolution. The skip connections connect corresponding layers of the encoder and decoder, enabling the decoder to access information from earlier stages of the network. Our network had six convolutional layers and five pooling layers, all with a kernel size of 3 × 3 × 3 for the convolutional layers and 2 × 2 × 1 for the first pooling layer followed by five 2 × 2 × 2.

R2-2

The network uses a combination of two loss functions, namely the dice loss and the cross-entropy loss (Supplementary Table 3). The initial network weights are initialized using the Kaiming He method, and the initial learning rate is set to 0.0003, which decays over time using the Adam optimizer [1]. nnU-Net uses various data augmentation techniques to increase the size and diversity of the training set. The augmentation techniques included rotation, gamma correction, scaling, elastic deformation, and mirroring. The input images were divided into patches of size 128 × 128 × 112, which were fed into the network during training. The batch size of 2 was relatively small as compared with other approaches, and it was chosen based on the available GPU memory. The patch and batch size were determined based on the size and resolution of the input images. The model was trained using Python version 3.7 and PyTorch version 1.2.

The conventional MR model performance converged after approximately 585 epochs of 38 hours by Adam optimizer with learning rate weight decay. The diagnostic performance of the combined MR model based on both the conventional MR imaging and V_e_ map converged after approximately 340 epochs of 23 hours (Supplementary Fig. 2).

R1-8

**Supplementary Tables**

**Supplementary Table 1.** Clinical characteristics of the recurrence and non-recurrence groups

| **Characteristics** | **Total**  **(n = 179)** | **Recurrence**  **(n = 76)** | **Non-recurrence**  **(n = 103)** | ***P* value** |
| --- | --- | --- | --- | --- |
| **Mean age (years)**^*^ | 57.7 ± 13.4 | 59.5 ± 12.7 | 56.4 ± 13.8 | 0.06^†^ |
| **Sex** |  |  |  | 0.13^‡^ |
| Male | 92 (51) | 45 (58) | 47 (46) |  |
| Female | 87 (49) | 32 (42) | 55 (54) |  |
| **Methylated MGMT promoter** |  |  |  | <0.001^‡^ |
| Positive | 98 (55) | 21 (27) | 77 (75) |  |
| Negative | 81 (45) | 56 (73) | 25 (25) |  |
| **IDH1/2 mutation** |  |  |  | 0.03^‡^ |
| Positive | 13 (7) | 2 (3) | 11 (11) |  |
| Negative | 165 (92) | 74 (96) | 91 (89) |  |
| Not available | 1 (1) | 1 (1) | 0 (0) |  |

Unless otherwise indicated, data represent the number of patients (percentages).

MGMT = O^6^-methylguanine-DNA methyltransferase, IDH = isocitrate dehydrogenase.

^*^ Data are means ± SD.

^†^ Calculated with the independent samples t-test.

^‡^ Calculated with Fisher’s exact test.

**Supplementary Table 2.** MRI scan parameters

|  | **Magnetom Verio** | **Magnetom Skyra** | **Ingenia CX 3.0T** | **Discovery MR 750w** |
| --- | --- | --- | --- | --- |
| Field strength (T) | 3.0 | 3.0 | 3.0 | 3.0 |
| Head coil channel | 32 | 64 | 32 | 32 |
| **T1WI** | | | | |
| TR (ms) | 1370 | 1670 | 8.1 | 7.5 |
| TE (ms) | 1.9 | 2.8 | 3.7 | 2.8 |
| FA (°) | 9 | 9 | 8 | 12 |
| NEX | 1 | 1 | 1 | 1 |
| Matrix | 256×232 | 256×232 | 240×240 | 256×256 |
| Section thickness (mm) | 1 | 1 | 1 | 1 |
| Intersection gap (mm) | 0 | 0 | 0 | 0 |
| FOV (mm) | 226×250 | 226×250 | 240×240 | 256×256 |
| **T2 FLAIR** | | | | |
| TR (ms) | 9000 | 8000 | 9000 | 8000 |
| TE (ms) | 97 | 105 | 100 | 89.4 |
| TI (ms) | 2800 | 2500 | 2500 | 2446 |
| FA (°) | 130 | 150 | 90 | 142 |
| NEX | 1 | 1 | 1 | 1.1 |
| Matrix | 384×184 | 384×244 | 380×243 | 288×288 |
| FOV (mm) | 175×220 | 199×220 | 219×219 | 220×220 |
| Section thickness (mm) | 5 | 4 | 4 | 5 |
| Intersection gap (mm) | 1.0 | 0.8 | 1.0 | 1.0 |
| **DCE MRI** | | | | |
| TR (ms) | 2.8 | 2.8 | 4.2 | 2.9 |
| TE (ms) | 1.0 | 1.0 | 2.1 | 1.1 |
| FA (°) | 10 | 10 | 10 | 10 |
| Matrix | 192×192 | 192×192 | 192×192 | 128×128 |
| FOV (mm) | 240×240 | 240×240 | 240×240 | 240×240 |
| Section thickness (mm) | 3.0 | 3.0 | 3.0 | 3.0 |
| Voxel size (mm^3^) | 1.25×1.25×3 | 1.25×1.25×3 | 1.25×1.25×3 | 1.87×1.87×3 |
| Pixel bandwidth (Hz) | 789 | 790 | 543 | 651 |
| Phase | 60 | 60 | 60 | 60 |
| Temporal resolution | 4.8 sec | 4.8 sec | 5.1 sec | 5−6 sec |
| Total acquisition time (sec) | 4 min 58 sec | 4 min 58 sec | 5 min 3 sec | 5 min 5 sec |

MRI = magnetic resonance imaging, T1WI = T1-weighted imaging, FLAIR = fluid-attenuated inversion recovery, TR = repetition time, TE = echo time, FA = flip angle, NEX = number of excitations, FOV = field of view, DCE MRI = dynamic contrast-enhanced MR imaging.

**Supplementary Table 3.** Parameters for the model training

| Parameter name | Value |
| --- | --- |
| Loss function | Dice and cross-entropy |
| Optimizer | Adam with learning rate weight decay |
| Initial Learning rate | 0.0003 |
| Weight initialization | Kaiming He |
| Data augmentation | Rotation, gamma, scaling, elastic deform, mirror |
| Batch size | 2 |
| Patch size | 128×128×112 |

**Supplementary Figures**

**
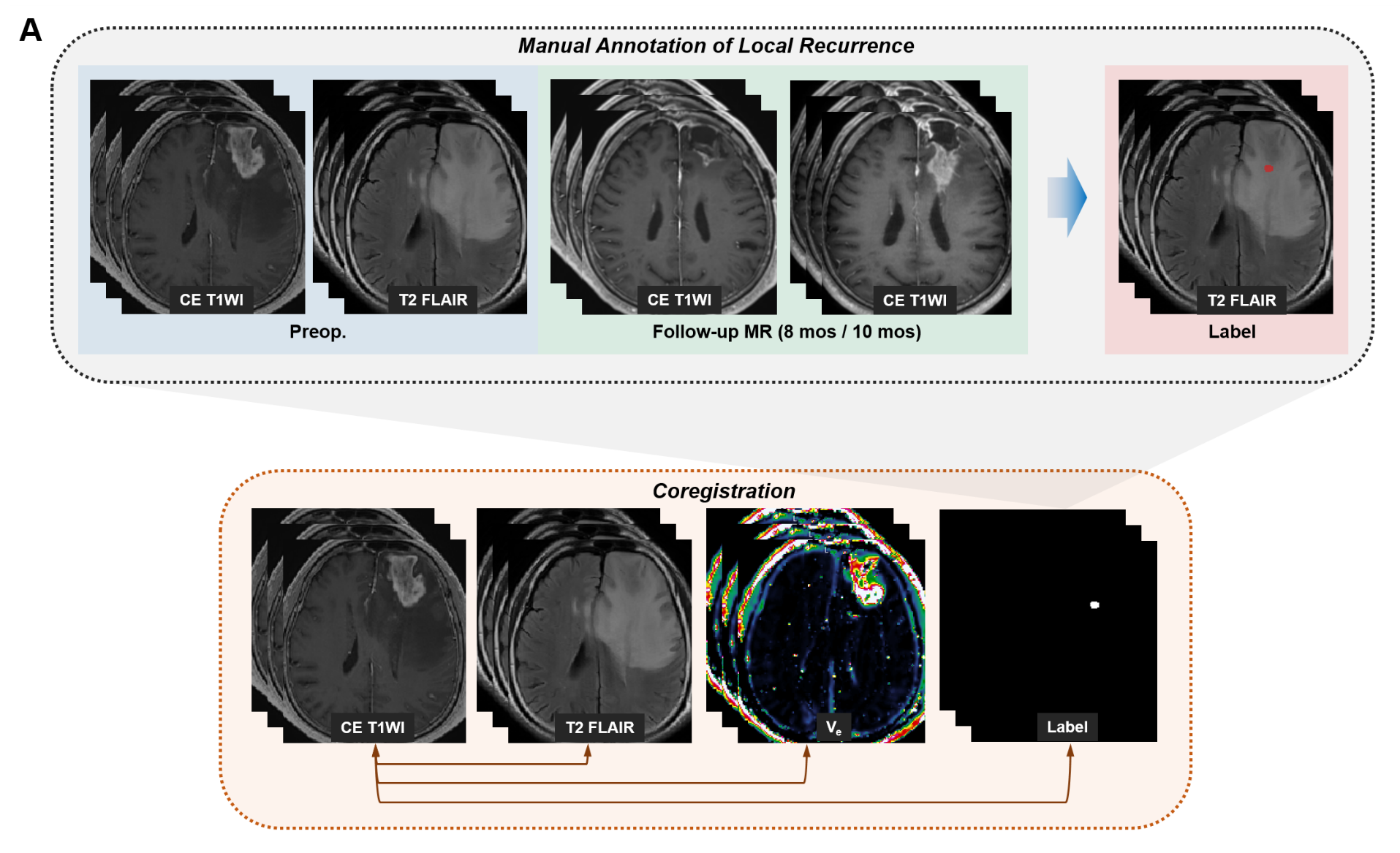
**

**
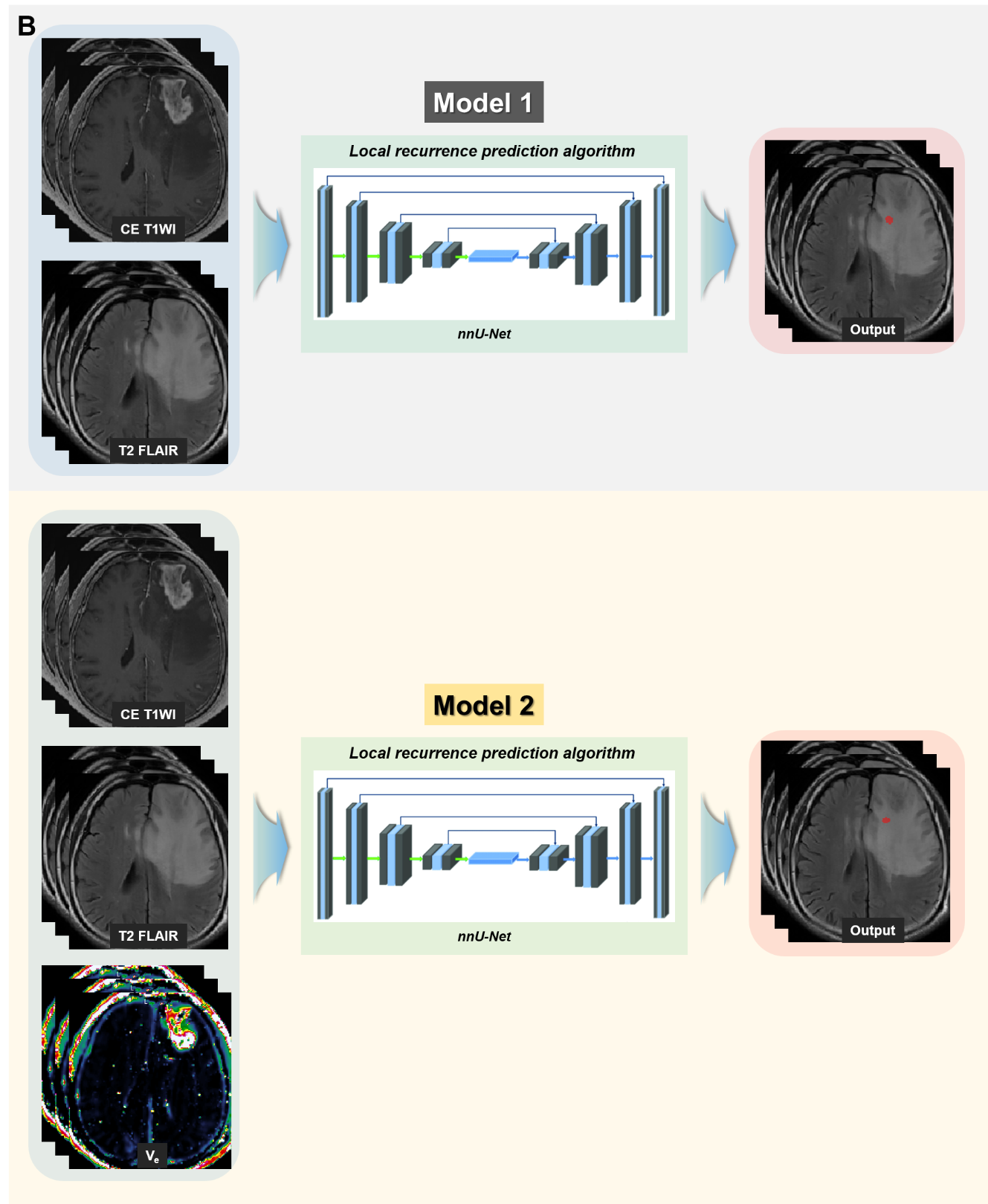
**

**Supplementary Figure 1.** Schematic workflow from data preprocessing to deep learning-based prediction of local recurrence. (A) Local recurrence site was manually labelled within every section of a non-enhancing T2 hyperintense lesion on preoperative FLAIR images using the follow-up CE T1W images as the reference standard. Preoperative T2 FLAIR images, V_e_ maps, and manual annotation labels were coregistered to the resampled CE T1W images. (B) Two nnU-Net based deep learning models were developed: ‘Model 1’ trained on conventional MR imaging alone and ‘Model 2’ trained on the combination of conventional MR imaging and V_e_ map. FLAIR = fluid-attenuated inversion recovery, CE T1W = contrast-enhanced T1-weighted.


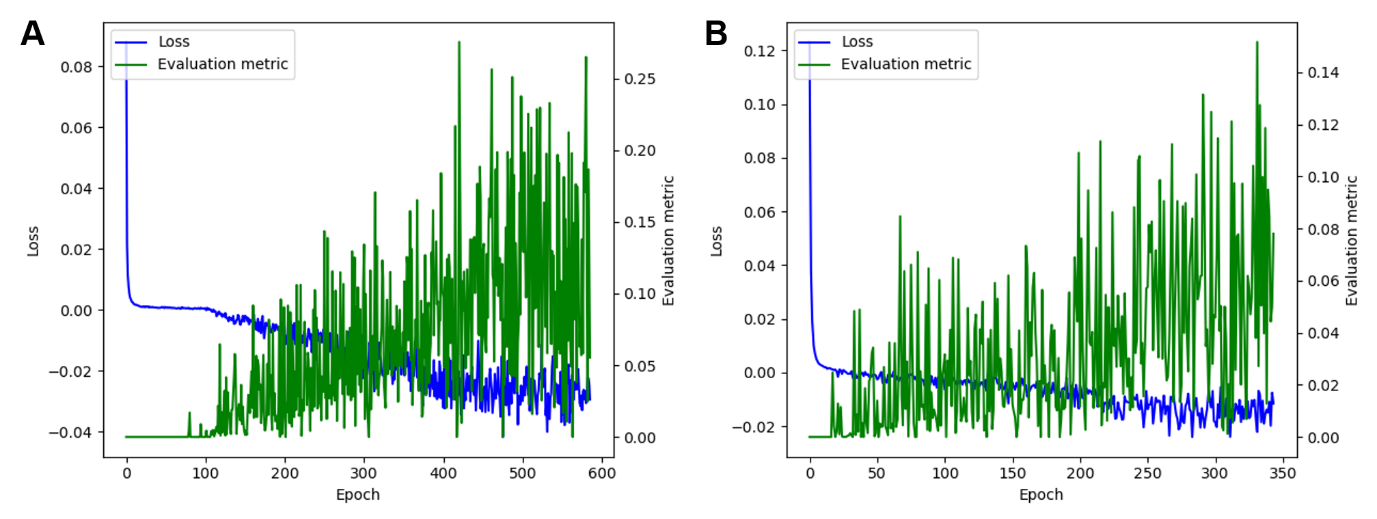


**Supplementary Figure 2.** Training curves for the deep learning models. With increasing iterations, the training models’ loss function steadily decreased while Dice score (moving averaged) steadily increased for both the conventional MRI model (A) and multiparametric MRI (B) model. Training terminated automatically after reaching the plateau at which the change in loss function (Dice loss + cross entropy) was minimal.

**References**

1 Kingma DP, Ba J (2014) Adam: A method for stochastic optimization. arXiv preprint arXiv:14126980
